# *In vivo* evidence of significant placental growth factor release by normal pregnancy placentas

**DOI:** 10.1038/s41598-019-56906-w

**Published:** 2020-01-10

**Authors:** Ana Sofia Cerdeira, Neva Kandzija, Pille Pargmae, Mariana Tome, Wei Zhang, William R. Cooke, Swati Agrawal, Tim James, Christopher Redman, Manu Vatish

**Affiliations:** 1 Nuffield Department of Women’s Health and Reproductive Research, University of Oxford, Level 3, Women’s Center, John Radcliffe Oxford University Hospital, Oxford, OX3 9DU United Kingdom; 2 Department of Obstetrics and Gynecology, Women’s Center, John Radcliffe Oxford University Hospital, Oxford, OX3 9DU United Kingdom; 3 Department of Biochemistry, John Radcliffe Oxford University Hospital, Oxford, OX3 9DU United Kingdom; 4 University of Toronto, Toronto, Canada

**Keywords:** Diagnostic markers, Pregnancy outcome

## Abstract

Placental growth factor (PlGF) is an angiogenic factor identified in the maternal circulation, and a key biomarker for the diagnosis and management of placental disorders. Furthermore, enhancing the PlGF pathway is regarded as a promising therapy for preeclampsia. The source of PlGF is still controversial with some believing it to be placental in origin while others refute this. To explore the source of PlGF, we undertook a prospective study enrolling normal pregnant women undergoing elective caesarean section. The level of PlGF was estimated in 17 paired serum samples from the uterine vein (ipsilateral or contralateral to the placental insertion) during caesarean section and from a peripheral vein on the same day and second day post-partum. PlGF levels were higher in the uterine than in the peripheral vein with a median difference of 52.2 (IQR 20.1–85.8) pg/mL p = 0.0006. The difference when the sampled uterine vein was ipsilateral to the placenta was 54.8 (IQR 37.1–88.4) pg/mL (n = 11) and 23.7 (IQR −11; 70.5) pg/mL (n = 6) when the sample was contralateral. Moreover, PlGF levels fell by 83% on day 1–2 post-partum. Our findings strongly support the primary source of PlGF to be placental. These findings will be of value in designing target therapies such as PlGF overexpression, to cure placental disorders during pregnancy.

## Introduction

There is discrepancy in the literature as to whether the placenta is a significant source of maternal circulating PlGF in normal pregnancies. *In vitro* studies have shown placental production and release of PlGF^1–3^. In fact, PlGF was first identified in 1991 by Maglione *et al*. in a placenta cDNA library^4^, hence its name. Since then however, PlGF has been found to be produced by malignant cells, endothelium, smooth muscle, pericytes, myocites and immune cells^5–7^. *In vivo* studies in pregnancy show conflicting data as to the primary source of PlGF^8–10^. The question remains as to how much maternal circulating PlGF is of placental origin.

Understanding PlGF biology in both healthy and diseased pregnancies is of major importance. PlGF plays a role in normal placental formation; changes in PlGF are associated with preeclampsia and adverse fetal outcomes^11–16^. Circulating PlGF forms a key component of novel preeclampsia biomarker assays recently introduced to clinical practice^17–19^. Strategies are currently being developed to enhance the PlGF pathway as a potential treatment for preeclampsia^20–22^; thus a better understanding of PlGF biodynamics will be crucial to designing the delivery mode and dosing.

Previous studies have examined *in vivo* PlGF placental production by interrogating the PlGF concentration gradient between the uterine vein (closer to placenta) and a peripheral vein. Whilst a difference has been shown by one group^8,10^, implicating the placenta as a main source of PlGF, this was not shown by another^9^. This discrepancy could be due to different methodologies used.

We aimed to determine if the placenta is the primary source of PlGF in normal pregnancies *in vivo*. We optimized previous conditions by using an automated assay with a low coefficient of variation, interrogating patients with lateral placentas, as well as making a comparison with postpartum peripheral levels.

## Methods

The study was conducted at the John Radcliffe Hospital, Oxford, United Kingdom. We recruited women admitted for an elective cesarean section. All pregnancies were uncomplicated and indications for caesarean section were previous caesarean section or breech presentation. sFlt-1 levels in this cohort have previously been reported^23^.

All women included in the study had an ultrasound scan to assess placental location prior to the elective caesarean section. Patients with a midline placenta or with any signs of early labour were excluded.

Serum samples were collected from the antecubital fossa (peripheral vein) and the uterine vein (UV) during the caesarean section, prior to delivery of the fetus. Uterine veins were identified laterally to the uterus and exposed by gentle displacement of the uterus towards the midline. Patients had a second peripheral sample taken on day 1–2 postnatally.

Uterine vein samples were labelled as contralateral or ipsilateral depending on their location in relation to the placenta. In the majority of cases the surgeon collected one sample either from the ipsilateral or contralateral uterine vein, at their own description or depending on surgical accessibility. In three cases of extreme lateral placentas it was possible to take bilateral uterine vein samples. Extreme lateral placenta was defined as a placenta located mostly on one side of the uterus: lateral left: all or almost all the placenta is located to the left of the midline; lateral right: all or almost all the placenta is located to the right of the midline; Midline placenta was defined as a placenta with a similar percentage of tissue towards the left and the right of the midline.

Non-parametric analysis, Wilcoxon rank test, was used to assess differences between paired PlGF results and P < 0.05 was considered significant.

Samples were centrifuged within 3 hours of collection and frozen at −80C until analysis. PlGF was measured on a Roche e411 analyzer (Roche Diagnostics Limited, Burgess Hill, UK). Inter-assay percentage coefficient of variation was 3.0% at 106.1 pg/mL and 2.9% at 1068.5 pg/mL.

All patients provided informed written consent prior to the start of the study. The study was approved by the Central Oxfordshire Research Ethics Committee C (07/H0607/74) and was in accordance with the ethical standards of the institutional and/or national research committee and with the Helsinki Declaration and its amendments.

## Results

**Figure 1:**
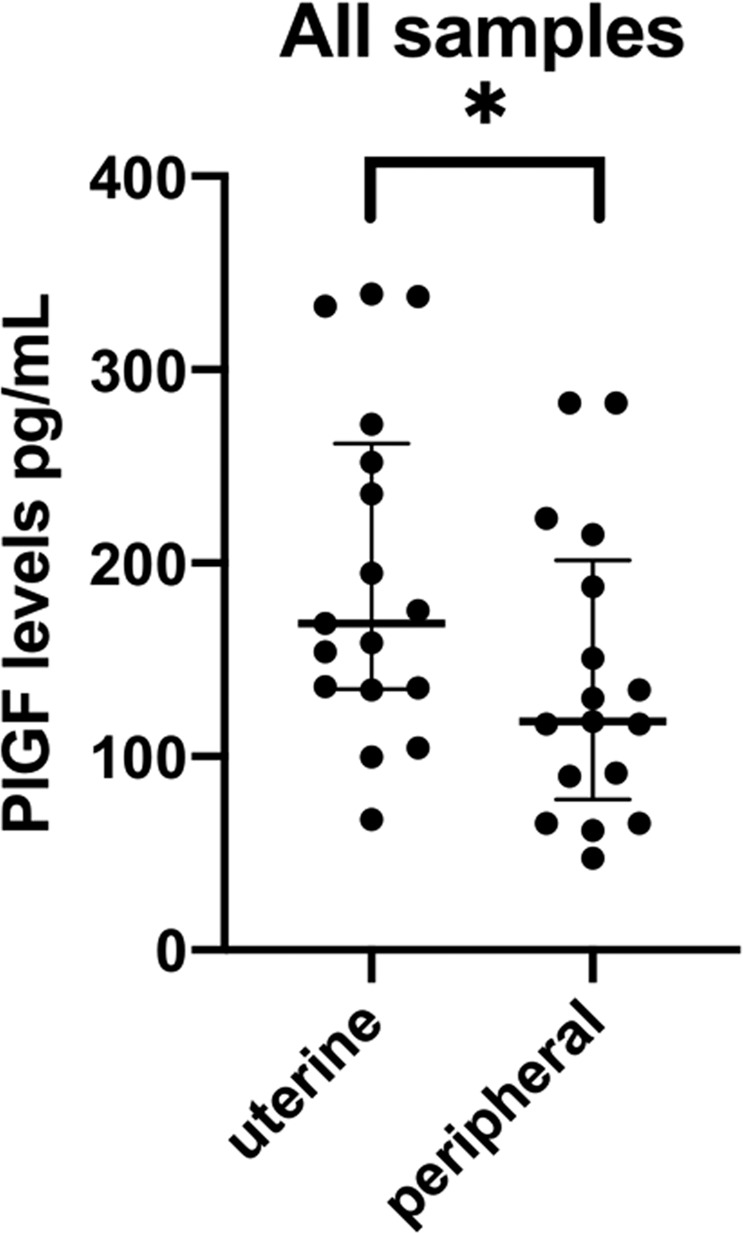
Placental growth factor levels for uterine and peripheral vein. Data is presented as individual values with median and interquartile range. Paired *Wilcoxon* test was performed. P < 0.05 was considered significant (*).

**Figure 2:**
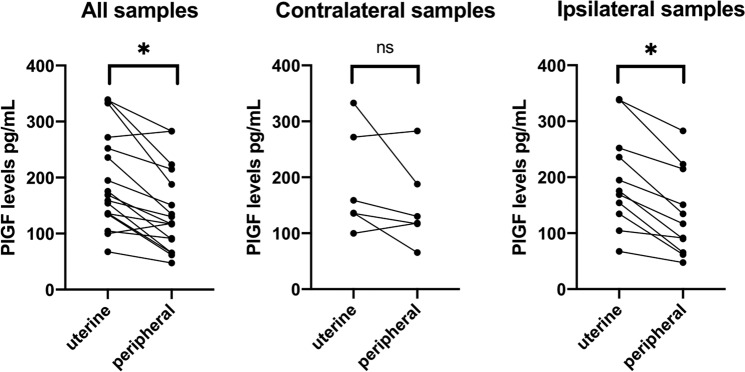
Placental growth factor levels for uterine and peripheral vein showing paired samples and placental location. (**A**) all paired samples are presented (n = 17 pairs); (**B**) contralateral samples are presented (n = 6 pairs). (**C**) Ipsilateral samples are presented (n = 11 pairs). Paired *Wilcoxon* test was performed. P < 0.05 was considered significant (*).

We obtained seventeen paired uterine and peripheral samples at the time of caesarean section. Mean gestational age was 39.4 (IQR 39.1–40) weeks. Overall median uterine vein PlGF was 168.9 (IQR 135.4–252) pg/mL and overall median peripheral PlGF was 118.2 (IQR 89.8–187.9) pg/mL (n = 17 paired samples, paired *Wilcoxon* test p = 0.0006) (Figs. 1 and 2).

**Figure 3:**
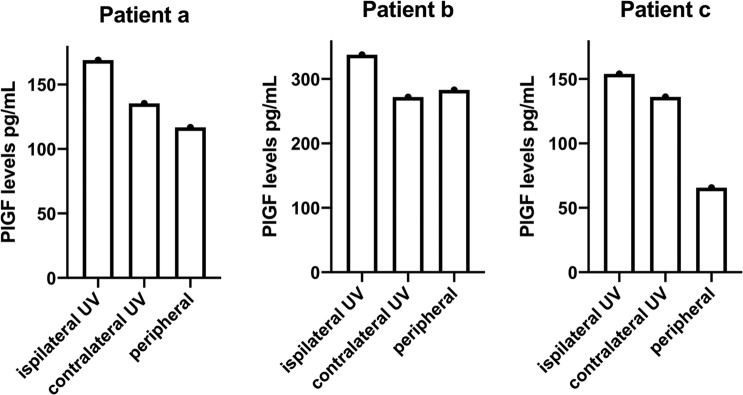
Placental growth factor levels for three patients with lateral placentas who had both (ipsilateral and contralateral) uterine vein samples collected. UV: uterine vein.

The median difference between PlGF uterine and peripheral vein concentrations was 52.2 (IQR 20.1–85.8) pg/mL, being 23.7 (IQR −11; 70.5) pg/ml when the UV sample was contralateral to the placenta (n = 6; paired *Wilcoxon* test p = 0.12) and 54.8 (IQR 37.1–88.4) pg/mL when the UV sample was ipsilateral to the placenta (n = 11; paired *Wilcoxon* test p = 0.003) (Fig. 2). Importantly, in 3 patients where bilateral UV samples were collected, PlGF levels from ipsilateral UV samples were consistently higher than contralateral ones (Fig. 3).

**Figure 4:**
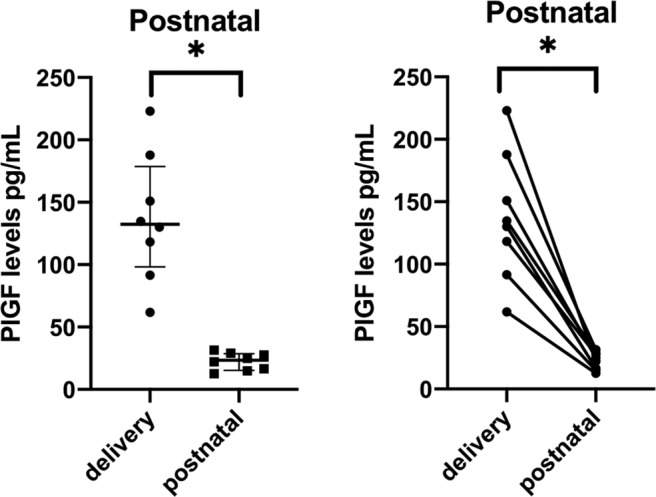
Placental growth factor levels at delivery and postnatal. Delivery stands for the peripheral samples collected at the time of caesarean section. Postnatal samples were collected at day 1–2 after delivery (Caesarean section) (n = 8). Data presented with all the samples all together and samples with their respective pairs; Paired *Wilcoxon* test was performed. P<0.05 was considered significant (*).

Peripheral PlGF levels fell by 83% postpartum [median PlGF: 23.5 (IQR 15.8–28.3); n = 8; paired *Wilcoxon* test p = 0.012] (Fig. 4).

## Discussion

PlGF is an angiogenic factor that belongs to the vascular endothelial growth factor (VEGF) family. It has an important role in normal placental function and can be detected in maternal circulation as early as the first trimester^24^. PlGF is tightly linked with the pathogenesis of preeclampsia and fetal growth restriction; thus understanding PlGF biodynamics will be crucial to making progress in therapeutics in these diseases. We demonstrate that the placenta is an important source of maternal circulating PlGF by showing a significant gradient between uterine and peripheral PlGF levels. This is most evident in uterine vein samples acquired from the same side as the placenta (ipsilateral). In support of this, bilateral samples collected from lateral placentas consistently showed higher PlGF levels in the ipsilateral uterine vein compared to the contralateral. Finally, when the placenta is removed (i.e. postnatal period) there is an 83% fall in PlGF levels in the peripheral circulation. It has been suggested that the uterus itself could be a key source of PlGF, rather than the placenta. *In vitro* studies showing significant production and expression of PlGF by normal placentas^1,25^ and our data from bilateral samples and postnatal samples (showing a significant drop of PlGF after the placenta is removed) suggests that this is unlikely. This study was performed on term patients and therefore the conclusions may not be applicable to other trimesters.

Most of our knowledge of placental biology is derived from human *in vitro* and *ex vivo* data, which is easier to access than *in vivo* samples, but remains a model. *In vitro* studies show that both VEGF and PlGF are produced by the placenta^25^. Intriguingly, in contrast with PlGF, *in vivo* data suggests that maternal circulating VEGF is taken up by the placenta and mostly produced by peripheral organs (shown by a lower concentration of VEGF in the uterine vein when compared to the radial artery)^10^. This not only demonstrates the value of *in vivo* data, but also has important implications. For example, when designing target-specific strategies, such as the recent use of RNAi to downregulate the production of sFlt-1 only in the placenta (the main source of sFlt-1) and not in other organs^26^.

In summary, these data strongly suggest that the placenta is the main source of maternal PlGF in normal pregnancy.
